# Neonatal inflammatory pain and systemic inflammatory responses as possible environmental factors in the development of autism spectrum disorder of juvenile rats

**DOI:** 10.1186/s12974-016-0575-x

**Published:** 2016-05-16

**Authors:** Jin Hwan Lee, Alyssa R. Espinera, Dongdong Chen, Ko-Eun Choi, Asha Yoshiko Caslin, Soonmi Won, Valentina Pecoraro, Guang-Yin Xu, Ling Wei, Shan Ping Yu

**Affiliations:** Department of Anesthesiology, Emory University School of Medicine, Atlanta, GA 30322 USA; Department of Neurology, Emory University School of Medicine, Atlanta, GA 30322 USA; The Laboratory of Translational Pain Medicine, Institute of Neuroscience, Soochow University, Suzhou, Jiangsu 215123 China; Center for Visual and Neurocognitive Rehabilitation, Atlanta VA Medical Center, Atlanta, GA 30033 USA; Emory University School of Medicine, 101 Woodruff Circle, WMB Suite 620, Atlanta, GA 30322 USA

**Keywords:** Inflammatory pain, Social behavior, Cell death, Autism spectrum disorder, NRXN1, FMR1, Oxytocin

## Abstract

**Background:**

Autism spectrum disorder (ASD) affects many children and juveniles. The pathogenesis of ASD is not well understood. Environmental factors may play important roles in the development of ASD. We examined a possible relationship of inflammatory pain in neonates and the development of ASD in juveniles.

**Methods:**

Acute inflammation pain was induced by 5 % formalin (5 μl/day) subcutaneous injection into two hindpaws of postnatal day 3 to 5 (P3–P5) rat pups. Western blot, immunohistochemical, and behavioral examinations were performed at different time points after the insult.

**Results:**

Formalin injection caused acute and chronic inflammatory responses including transient local edema, increased levels of inflammatory cytokines, TNF-α, and IL-1β in the blood as well as in the brain, and increased microglia in the brain. One day after the pain insult, there was significant cell death in the cortex and hippocampus. Two weeks later, although the hindpaw local reaction subsided, impaired axonal growth and demyelization were seen in the brain of P21 juvenile rats. The number of bromodeoxyuridine (BrdU) and doublecortin (DCX) double-positive cells in the hippocampal dentate gyrus of P21 rats was significantly lower than that in controls, indicating reduced neurogenesis. In the P21 rat’s brain of the formalin group, the expression of autism-related gene neurexin 1 (NRXN1), fragile X mental retardation 1 (FMR1), and oxytocin was significantly downregulated, consistent with the gene alteration in ASD. Juvenile rats in the formalin group showed hyperalgesia, repetitive behaviors, abnormal locomotion, sleep disorder, and distinct deficits in social memory and social activities. These alterations in neuroinflammatory reactions, gene expression, and behaviors were more evident in male than in female rats. Importantly, an anti-inflammation treatment using indomethacin (10 mg/kg, i.p.) at the time of formalin injections suppressed inflammatory responses and neuronal cell death and prevented alterations in ASD-related genes and the development of abnormal behaviors.

**Conclusions:**

These novel observations indicate that severe inflammatory pain in neonates and persistent inflammatory reactions may predispose premature infants to development delays and psychiatric disorders including ASD. The prevention of pain stimuli and prompt treatments of inflammation during development appear vitally important in disrupting possible evolution of ASD syndromes.

**Electronic supplementary material:**

The online version of this article (doi:10.1186/s12974-016-0575-x) contains supplementary material, which is available to authorized users.

## Background

In the USA, one out of eight infants is born premature [1]. Preterm infants are at increased risk for developmental disorders, abnormal behaviors, and cognitive dysfunction syndromes that can be associated with autism spectrum disorder (ASD) [2, 3]. ASD includes a heterogeneous group of early onset childhood neurodevelopmental disorders [4]. The prevalence of ASD is 1 in 150 individuals and occurs more frequently in males than in females [5]. The incidence estimation may be even higher, affecting 1 in 68 children when combining three basic categories of neurodevelopmental disorders: autistic disorder (autism), Asperger syndrome, and pervasive developmental disorder (PDD-NOS) [6]. Although the clinical symptoms are heterogeneous, ASD patients show common characteristics including social interaction deficits, communication difficulties, stereotyped repetitive behaviors, limited repertoire of interests, and, in some cases, cognitive problems [7]. Early symptoms of ASD may include locomotion impairment [7, 8]. ASD patients may suffer from comorbid conditions such as anxiety, epilepsy, intellectual disability, and depression [8]. According to the recent guideline of American Psychiatric Association (APA), clinical diagnostic criteria include the following: (a) persistent deficits in social communication and social interaction across multiple contexts; (b) Restricted, repetitive patterns of behavior, interests, or activities; (c) symptoms must be present in the early developmental period; (d) symptoms cause clinically significant impairment in social, occupational, or other important areas of current functioning; and (e) these disturbances are not better explained by intellectual disability (intellectual developmental disorder) or global developmental delay [9]. In this report, we used the term autism spectrum disorder or ASD to refer to autism-like behaviors in the animal model tested in this investigation.

The contribution of genetic and environmental factors to the development of ASD has drawn increasing attention in basic and clinical research. ASD most likely emerges from a complex interaction between pre-existing genetic vulnerabilities and environment factors. Although a number of genes such as neurexin 1 (NRXN1), fragile X mental retardation 1 (FMR1), and oxytocin/oxytocin receptors have been identified to be ASD related, the contribution of environmental factors to the development of ASD is not well understood. It was noticed that children born prematurely more often display poorer executive functionality and cognition and are more likely to have behavioral problems [10]. There are reports that premature infants under stress or surgery may show increased inflammatory factors such as TNF-α and IL-6 [11–13]. It was suggested that premature birth and susceptibility genes may make infants more vulnerable to allergic, environmental, infectious, or stress-related triggers that could stimulate mast cell release of pro-inflammatory and neurotoxic molecules, thus contributing to brain inflammation and ASD pathogenesis [12]. In clinical practices, premature infants in the neonatal intensive care unit (NICU) are routinely exposed to an average of ten therapies or procedures per day without analgesics [14]. Many of the procedures are painful and may cause inflammatory responses and local tissue edema/damage. It is now believed that infants are more sensitive to pain due to the incomplete development of the brain and the descending inhibitory tracts in their spinal cord [15]. Further, the immature sensory processing system within the newborn spinal cord results in lower thresholds for excitation and sensitization [16]. Although a few reports noticed different pain experiences in ASD children and discussed special care for these young patients [17, 18], the possibility that early life inflammatory pain experience influences the progression of ASD has not been explored.

A subcutaneous formalin injection-induced acute inflammatory pain model has been widely used for many years in pain research [19, 20]. Formalin-produced local response patterns lasting for approximately 1 h are composed of phase I reactions for about 5 min followed by a longer phase II reaction of about 40 min, characterized by shaking and/or linking of the paw(s). This model is suitable for the examination of acute inflammatory pain and the chronic consequences following the acute pain insult. In the present investigation, the inflammatory pain insult was applied to postnatal day 3 to 5 (P3–P5) pups that are equivalent in brain developmental stage to human preterm infants [21]. Using this model, we aimed to elucidate whether repeated inflammatory pain experienced by preterm/premature babies could lead to acute and delayed brain damage that might be associated with social and behavioral abnormalities at the juvenile age.

## Methods

### Animals and ethics, consent, and permissions

Wistar rats (female adult mothers, neonatal pups, and juvenile rats of male and female sex) were kept in the Emory University animal facility under environmental control of standardized room temperature (22–23 °C), low humidity, and 12-h lighting circle. Animals were allowed free access to water and food. Postnatal rat pups stayed with their mothers during experimental periods. All studies were approved by the Institutional Animal Care and Use Committee (IACUC) at Emory University.

### Inflammatory pain model of neonatal rats

A subcutaneous injection (sc) of formalin induces progressive and selective activation of the somatosensory pathway and limbic system structures in the brain and brain stem [20]. Formalin injection causes a biphasic response. The early phase (0–5 min) results mainly from C-fiber activation due to the peripheral stimulus, while the late phase (more than 20 min) results from the combination of an inflammatory reaction in the peripheral tissue and functional changes in the dorsal horn of the spinal cord [19, 20].

The inflammatory pain model followed our previous procedures with some modifications [22]. Male and female rat pups at postnatal day 3 (P3) received 5-μl subcutaneous injections of 5 % formalin or saline solution, to each hind paw. The second paw injection was performed 1 h after the first one to give the pups some rest. Ten-μl Luer lock syringes (Hamilton Co., Reno, NV) fitted with an intradermal needle were used for injections. For response to the inflammatory pain, animals were sacrificed 24 h after these formalin injections. For sub-acute and chronic consequences of the pain insult, two more injections were performed at P4 and P5 and sacrificed at different specified times later. After each injection, the pups were immediately returned to their mothers in the home cage.

### Drug administration

Indomethacin was purchased from Sigma. Indomethacin (10 mg/kg) was administered intraperitoneally within 10 min after injections of 5 % formalin or saline solution.

### Paw volume measurements

The paw volume of rats was measured 1 day before the first formalin injection and 1,3, 5, 7, 9, 11, 13, and 18 days after the first formalin injection using a plethysmometer (UGO Basile, Varese, Italy). For each day, the edema was expressed as the increase in paw volume, and the percentage of induction of edema was expressed as the increase in volume with respect to the control group.

### Immunohistochemistry

After sacrifice and dissection, brains were immediately frozen at −80 °C in the optimal cutting temperature (OCT) compound (Tissue-Tek®, Sakura Finetek USA, Inc., Torrance, CA). Sections were cut at 10-μm thickness using a cryostat (Leica Biosystems, Buffalo Grove, IL). Brains from animals that were perfused with 0.9 % saline (pH 7.4) followed by 10 % buffered formalin for detection of myelin basic protein (MBP) and bromodeoxyuridine (BrdU) immunoreactivities were removed and placed in formalin for 24 h, then placed in a 30 % sucrose solution at −20 °C in OCT compound, and were cut into 14-μm thick sections on a cryostat (Leica Biosystems).

The brain sections were then fixed for 10 min in 10 % buffered formalin, washed in phosphate buffered saline (PBS) three times, then incubated in −20 °C ethanol acetic acid (2:1) for 5 min or methanol for 7 min. Sections were washed in PBS three times and then incubated in 0.2 % TritonX-100 for 5 min. After three more washes in PBS, sections were incubated in 1 % fish gelatin (Sigma, St. Louis, MO) for 60 min. Sections were again washed in PBS or automation buffer, and an appropriate primary antibody was applied for overnight incubation at 4 °C: anti-neuronal nuclei (NeuN; Millipore, Billerica, MA), anti-neurofilament (NF; Millipore), anti-BrdU (AbD Serotec; Raleigh, NC or Santa Cruz Biotechnology, Dallas, TX), anti-MBP (Millipore), anti-neurokinin 1 receptor (NK-1R; Millipore), and anti-Iba-1 (Biocare Medical, Concord, CA). Slides were then washed and incubated with the appropriate conjugate secondary antibody for 60 min at 37 °C: donkey anti-mouse Cy5, donkey anti-rat and donkey anti-rabbit Cy3 (Jackson ImmunoResearch, West Grove, PA), and donkey anti-goat 488 (Invitrogen, Grand Island, NY). In some slides, nuclei were counterstained with Hoechst 33342 (1:20,000; Molecular Probes, Eugene, OR) for 5 min. Slides were washed three times in PBS and cover-slipped prior to imaging under a fluorescent microscope (Olympus BX61; Olympus America, Inc., Melville, NY). The image data were collected using the SlideBook 4.2 software (Olympus America, Inc.). All measurements were performed by an individual who was blinded to the experimental groups.

### TUNEL staining

In brain section containing the cortex, hippocampus, and other regions, terminal deoxynucleotidyl transferase dUTP nick end labeling (TUNEL) staining was performed using a commercial kit (DeadEnd™ Fluorometric TUNEL system; Promega, Madison, WI) to label DNA fragmentation in dead or dying cells in brain regions. In brief, brain sections were placed in equilibration buffer and incubated with nucleotide mix and rTdT enzyme at 37 °C for 1 h and 15 min. Reactions were terminated by ×2 SSC solution for 15 min. Nuclei were counterstained with Hoechst 33342 (1:20,000; Molecular Probes) for 5 min. TUNEL-positive cells were visualized using the fluorescein isothiocyanate (FITC) channel on the Olympus fluorescence microscope (Olympus America, Inc.).

### Cell counting

Cell count was performed following the principles of design-based stereology. Systematic random sampling was employed to ensure accurate and non-redundant cell counting. Every section under analysis was at least 100 μm away from the next. Six 10- to 14-μm thick sections, frozen or perfusion fixed, spanning the entire region of interest, were selected for cell counting. Counting was performed on six non-overlapping randomly selected ×20 fields per section. Images were taken in an anterior to posterior direction from the same region of the cortex defined according to a standard atlas of the rat brain. Cell counting was performed by an individual who was blinded to the experimental groups.

The data analysis of reactive microglia in the brain regions was based on the morphological assessment of Iba-1-positive cells according to published method [23]. Briefly, based on the length of branches, thickness of branches, and cell body volume, the Iba-1-positive cells were categorized to three classes: (a) ramified microglia (surveillant/resting microglia), characterized by small round or oval cell bodies containing a small volume of cytoplasm; (b) hypertrophied microglia, which had larger cell bodies and thicker processes than ramified microglia; and (c) bushy microglia, which had numerous but short processes forming thick bundles around their swollen cell bodies. Hypertrophied and bushy Iba-1-positive cells were identified as activated microglia.

### Axon measurements

To study axon diameter and distribution, a minimum of 100 axons labeled by neurofilament (NF) were randomly selected per brain section. Three sections were randomly selected for each animal, and 100 axons per section were analyzed in cortical areas adjacent to layer VI, aligned radially, and perpendicular to the cutting field. Axonal diameter was estimated by measuring the diameter perpendicular to the center of the maximum diameter of the axon profile, as previously described by Zikopoulos and Barbas [24]. Measured axons were then categorized as small (<0.35 μm), medium (0.35–0.69 μm), large (0.7–1.4 μm), and extra-large (>1.4 μm).

Myelinated axons were quantified using the principles of design-based stereology stated above. Axons labeled with MBP were quantified using ImageJ software (NIH, Bethesda, MD, USA) area fraction measurements to determine the density of myelinated axons in the region of interest. Counting was performed on six non-overlapping randomly selected ×20 fields per section. Each section was 14-μm thick and at least 100 μm from the next section.

### Isolation of total RNA and quantitative RT-PCR

Total RNA was extracted from the whole blood and the specific brain regions of rats in formalin and control groups using RiboPure™-Blood Kit (Invitrogen) and TRIzol reagent (Invitrogen), respectively. RNA integrity was confirmed by the detection of 28s and 18s rRNA bands in 1 % agarose gel with ethidium bromide. Also, RNA was confirmed to be free of genomic DNA contamination by PCR in the absence of reverse transcriptase. The RNA samples were reverse transcribed in 20 μl of a reaction mixture containing ×2 RT buffer and ×20 RT enzyme mix according to the manufacturer’s instructions (Life Technologies, Grand Island, NY, USA) at 37 °C for 60 min. The samples were then incubated at 95 °C for 5 min and transferred to 4 °C. For measuring gene expressions, quantitative real-time polymerase chain reaction (qRT-PCR) was done with an ABI 7500 Fast Real-Time system (Applied Biosystems, Foster City, CA, USA) with the FastStart DNA Master SYBR Green kit (Roche Diagnostics, Mannheim, Germany), and results were analyzed with the 7500 software supplied with the machine. GAPDH was used as an internal control. PCR primers used were listed as follows: for TNF-α, 5′-ATGGCCTCCCTCTCAGTTC-3′ (forward) and 5′-TTGGTGGTTTGCTACGACGTG-3′ (reverse); for IL-1β, 5′-CATCTTTGAAGAAGAGCCCG-3′ (forward) and 5′-AGCTTTCAGCTCACATGGGT-3′ (reverse); for IL-6, 5′-GCCCTTCAGGAACAGCTATG-3′ (forward) and 5′-CGGACTTGTGAAGTAGGGA-3′ (reverse); for substance P (SP), 5′-ATGAAAATCCTCGTGGCGGT-3′ (forward) and 5′-CAGCATCCCGTTTGCCCATT-3′ (reverse); and for 18s, 5′-ACCACAGTCCATGCCATCAC-3′ (forward) and 5′-CACCACCCTGTTGCTGTAGCC-3′ (reverse).

### Western blot analysis

Western blotting was used to detect the expression of inflammatory- and ASD-related genes. After sacrifice, animals were subjected to transcardial perfusion using PBS. Brain cortical and hippocampal tissues were lysed in a buffer containing 0.02 M Na_4_P_2_O_7_, 10 mM Tris-HCl (pH 7.4), 100 mM NaCl, 1 mM EDTA (pH 8.0), 1 % Triton, 1 mM EGTA, 2 mM Na_3_VO_4_, and a protease inhibitor cocktail (Sigma). The supernatant was collected after centrifugation at 15000*g* for 10 min at 4 °C. Protein concentration was determined with a bicinchoninic acid assay (Pierce Biotechnology, Rockford, IL, USA). Equivalent amounts of total protein were separated by molecular weight on an SDS-polyacrylamide gradient gel and then transferred to a PVDF membrane. The blot was incubated in 10 % nonfat dry milk for 1 h and then reacted with primary antibodies at 4 °C for overnight.

The primary antibodies and their dilutions are as follows: rabbit anti-TNF-α antibody (Cell Signaling) 1:2000, rabbit IL-1β antibody (Cell Signaling, Danvers, MA, USA) 1:1000, rabbit IL-6 antibody (Cell Signaling) 1:1000, mouse anti-actin (Sigma) 1:5000, rabbit anti-NK-1R (Millipore) 1:2500, mouse anti-neurexin 1 (NRXN1; Cell Signaling) 1:1000, rabbit anti-fragile X mental retardation 1 (FMR1; Cell Signaling) 1:1000, mouse anti-neuroligin3 (NLGN3; Millipore) 1:1000, rabbit anti-autism susceptibility gene 2 (AUTS2; Abcam) 1:2000, goat anti-oxytocin (Abcam) 1:1000, and rabbit anti-oxytocin receptor (Santa Cruz) 1:500. After washing with Tris-buffered saline with Tween-20 (TBST), membranes were incubated with AP-conjugated or HRP-conjugated secondary antibodies (GE Healthcare, Piscataway, NJ, USA) for 2 h at room temperature. After final washing with TBST, the signals were detected with bromochloroidolylphosphate/nitroblue tetrazolium (BCIP/NBP) solution (Sigma) or film. Signal intensity was measured by ImageJ (NIH) and normalized to the actin signal intensity.

### Behavioral tests

#### Locomotor activity using TopView system

Behavioral changes of experimental rats were monitored and analyzed using the TopScan System (Clever Sys Inc., Reston, VA, USA). P10, P15, and P20 rats were allowed to freely move in an open field container (50 cm × 50 cm × 50 cm high) during the dark cycle. Travelled distance and velocity for locomotor activity were recorded for 1 h. After finishing the recording, the videos were analyzed by the TopScan Realtime Option Version 3.0 (Clever Sys Inc.).

#### Hot-plate test

Pain sensitivity was measured using a hot-plate set to 55 +/− 1 °C. Response latency was measured as the time taken for the rat to jump after placing on the hot plate. The maximum allowed time was 30 s. The reported latency was the average value calculated from three measurements per animal. Repeated tests were separated by at least 15 min.

#### Three chamber sociability test

The three-chamber test was utilized to test general sociability and response to social novelty. The test was performed in a three-chambered box that has openings between the chambers. Glass slides were used to cover the openings during phase changes. First, the test subject was placed into the empty box and allowed to explore all chambers freely for 10 min. After the habituation period, a stranger (non-littermate) rat contained in a wire cage was placed into the left chamber. The rat was then allowed to explore all three chambers. Both the time spent with the stranger rat (stranger #1) and the time in the empty chamber were recorded over a 10-min session. The test rat was then returned to the center chamber, and the openings were blocked. In the social novelty test, a second stranger rat (stranger #2) was placed in the empty chamber. The central chamber door was opened, and the test rat was free again to explore the strangers #1 and #2. Since the test rat had already had contact with stranger #1 but not #2, the time it spent with strangers #1 and #2 tested its novel social interaction.

#### Social interaction test

This test was conducted with untreated, unfamiliar, weight-matched partner same sex rats. Subject and stranger rats were put together in a clean empty cage and recorded by the TopScan System (Clever Sys Inc.). We scored time spent in social interaction (social sniffing, social grooming, and social following) of the animals for 5 min between 1:00 and 5:00 pm. The cage was washed with a 70 % alcohol solution and water before we performed the next test in order to prevent possible contamination by previous tests.

#### Home cage observation

The HomeCage Monitoring System (Clever Sys Inc.) was used to detect the behavior patterns of animals in their home cage environment without human intervention. The system had four cameras that monitor four rats simultaneously in four separate cages (191 mm x 292 mm x 127 mm). Empty cages were first recorded and saved as the background image for video analysis. Animals were placed one per cage and allowed to habituate to the new environment for at least 30 min. The behavior patterns were recorded from 10 pm to 4 am during the night time when rodents are most active. The video recordings were analyzed using the HomeCage Software 3.0 (Clever Sys Inc.). The software discriminates various body movements and behavior patterns. We analyzed both the number of bouts of each behavior and the time spent performing each behavior during the 6-h period.

#### Morris water maze test

The Morris water maze test was performed and analyzed to measure memory function [25]. This test was videotaped using TopScan (Clever Sys, Inc.), and performance was analyzed using TopScan Realtime Option Version 3.0 software (Clever Sys, Inc.). The water maze apparatus is a round, water-filled tub (120.1-cm diameter filled with blue tempera paint) placed in a room rich with extra-maze cues. Rats were placed in the maze starting from four different positions (NW, NE, SW, and SE). An invisible escape platform was located in the same spatial location 1 cm below the water surface independent of the starting position on a particular trial. In this manner, subjects were able to utilize extra-maze cues to determine the platform’s location. Each subject was given four trials per day (NW, NE, SW, and SE) for 6 days with a 15-min inter-trial interval. The maximum trial length was 60 s, and subjects were manually guided to the platform if they did not reach it in the allocated time. Upon reaching the invisible escape platform, subjects were kept on it for an additional 15 s to allow them to survey the spatial cues in the environment to guide future navigation to the platform. After 6 days of task acquisition, a probe trial was carried out during which the platform was removed. The time spent and distance travelled in the quadrant that previously contained the escape platform during task acquisition was measured over 60 s.

#### Social transmission of food preference transmission test

The social transmission of food preference test was used in rodents to assess olfactory memory processes. For the test, two demonstrator rats were removed from each test cage and individually housed overnight with water but without food (18 h). The demonstrator rats were then placed into clean cages containing almond-flavored food in small glass jars (3.9-cm diameter, 3.4-cm high). The glass jars were set in shallow Petri dishes so that food scattered by the digging of the rats was retained. Demonstrator rats were left to eat the cued food (almond) for 1 h. Dishes were weighed before and after to measure how much food was eaten. The demonstrator rat was then placed in a clean experimental cage. One at a time, “observer” rats from the same home cage of the demonstrator rat were placed in the cage containing the demonstrator rat and left there for 5 min. The observer rat was then removed. After an interval of 15 min, the sequence of interaction was repeated, with each observer rat being placed with the second demonstrator rat from the home cage. All observer rats were then returned to the home cage, and demonstrator rats were individually housed. Six hours after the social interaction sessions, the observer rat was food-deprived for 18 h (overnight). The following morning, each rat was placed individually in a clean cage (45 cm × 28 cm × 12 cm) containing two “dishes” in either corner at the back of the cage: one with almond-flavored food (cued) and the other containing normal diet (non-cued). Rats were allowed to eat for 1 h. Dishes were weighed before and after to determine the amount of food eaten. Food preference was calculated as the amount of cued food eaten/total food eaten ×100 (% total).

#### Direct interaction test

To measure the social memory function, we performed the direct interaction test as described previously [26]. In the first trial, subject rats were placed in a clean cage, and a novel rat was introduced. Social interaction activity was quantified to examine the time spent in social sniffing, social following, and social grooming. After an inter-trial interval of 1 h, either the previously encountered rat or novel rat was introduced and then the social interaction activity was measured for 5 min.

#### Five-trial social memory test

The five-trial social memory test was performed to measure more obvious social memory ability as described previously [26]. Briefly, subject rats were presented with four successive 1-min trials. On the last trial, we introduced a novel rat. In each trial, we measured the social interaction activity (nose-to-nose sniffing, following, and grooming).

### Statistical analysis

All analyses were performed using GraphPad Prism 6.0 statistical software (GraphPad Software, Inc., La Jolla, CA). Multiple comparisons were performed by one- or two-way analysis of variance (ANOVA) followed by Bonferroni’s post hoc analysis. Single comparisons were performed using Student’s *t* test. Changes were considered significant if the *P* value was less than 0.05. Mean values were reported with the standard error of the mean (SEM) unless otherwise indicated.

## Results

### Local reactions of formalin-induced peripheral inflammatory pain in neonatal rats

Subcutaneous injection of 5 μl 5 % formalin or saline was applied to right and left hindpaws of postnatal day 3 (P3) rats, followed by two additional injections at postnatal days 4 and 5, respectively. These repeated injections were to mimic acute insults experienced by human premature babies subjected to multiple procedures in NICU. Rat pups in the formalin group showed hindpaw local edema and redness that reached to peak levels at P8. The formalin-induced edema progressively subsided thereafter and completely dissipated by P16 (Additional file 1: Figure S1A and S1B). Using a TopScan system analysis, we observed that P10 and P15 rats that received formalin injections travelled less distance and showed a lower velocity than control rats (Additional file 1: Figure S1C and S1D). The low locomotion completely disappeared at P20 (Additional file 1: Figure S1C and S1D). Considering the prevalence of ASD is higher in male than in female, different sex groups were analysis and compared in many experiments of this investigation. The locomotor deficits were seen with both male and female rats (Additional file 1: Figure S1C and S1D). Sexual dimorphism in the examination of general condition such as fur density/color and eating activity did not show significant difference. The body weight underwent a trend of slower gain within 10 days after formalin insult in both male and female but gradually caught up in following days (Additional file 2: Table S1)

### Peripheral inflammatory pain-induced acute and chronic increases of inflammatory cytokines in the blood circulation and brain

**Fig. 1:**
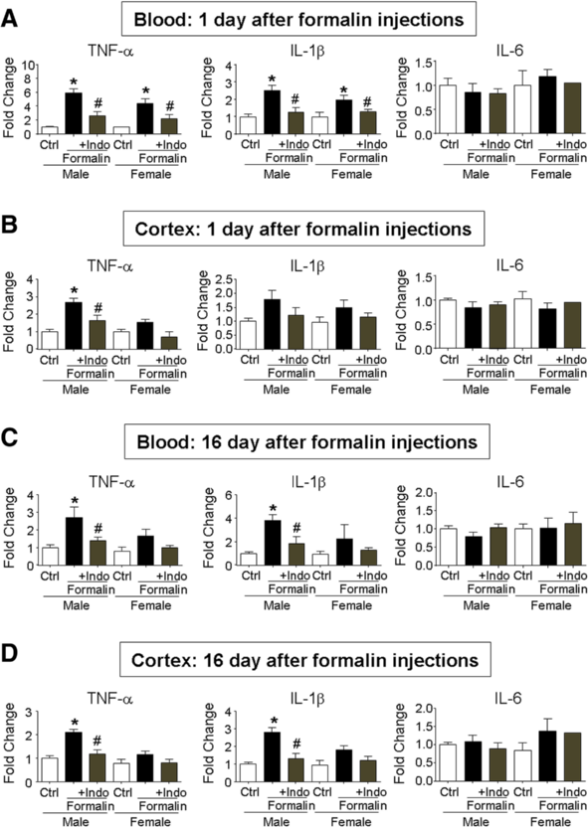
Neonatal inflammatory pain increased inflammatory cytokines in the blood and cortex. Quantitative RT-PCR (qRT-PCR) analysis was performed to measure inflammatory factors in blood samples and cortical tissues. **a** The levels of TNF-α, IL-1β, and IL-6 in the blood 1 day after the 3-day formalin injections, TNF-α and IL-1β significantly increased in both male and female rats; indomethacin (Indo) co-applied with formalin blocked the inflammatory reaction. **b** TNF-α, IL-1β, and IL-6 levels in the cortex 1 day after formalin injection. Only the TNF-α level was significantly enhanced in the male brain. Indomethacin (Indo) showed inhibitory effect on TNF-α expression. **c** Inflammatory factors were measured in blood samples 16 days after formalin injections (P21 rats). TNF-α and IL-1β increased in male but not in female rats in the formalin group, which were blocked by indomethacin (Indo). **d** Increased levels of TNF-α and IL-1β persisted in the male brain cortex 16 days after formalin injections. Indomethacin (Indo) blocked these increases. *P < 0.05 vs. control, ^#^ *P* < 0.05 vs. formalin group, ANOVA plus Bonferroni’s correction; *n* = 4 per group

To determine whether the local acute inflammatory pain could trigger systemic and lasting inflammatory responses, we measured inflammatory cytokines in the blood and brain cortical tissues. One day after the 3-day formalin insult, quantitative real-time polymerase chain reaction (qRT-PCR) assays showed significant increases of tumor necrosis factor alpha (TNF-α) and interleukin-1 beta (IL-1β) in the blood both in male and female rats (Fig. 1a). No significant change was seen in the level of IL-6 (Fig. 1a). At the same time, the TNF-α level in the brain cortex significantly increased in male rats but not in female rats (Fig. 1b). No significant increase in IL-1β and IL-6 was seen at this time in the brain of either sex group (Fig. 1b). Interestingly, chronic overexpression of TNF-α and IL-1β was detected in both the blood and cortex of P21 male rats, i.e., 16 days after the formalin insult (Fig. 1c, d). The increases were not detected in female rats. To block formalin-induced inflammation, a separate group of rats received anti-inflammatory pain treatment by the nonsteroidal drug indomethacin (10 mg/kg, i.p.) co-administered with formalin. This treatment prevented all acute and chronic increases of inflammatory factors in the blood and brain (Fig. 1a–d).

Substance P is an important element in pain perception, related to the transmission of pain information into the central nervous system. Substance P can be increased acutely but decreased after repetitive pain stimuli [27, 28]. We noticed a significant and chronic reduction in substance P level in the P21 male brain and indomethacin co-applied with formalin prevented this alteration (Additional file 3 Figure S2A-S2B). Neurokinin 1 receptor (NK-1R) is a G-protein-coupled substance P receptor found in the nervous system and highly expressed in the hippocampus [28]. In immunohistochemical staining assays, fewer NK-1R-positive cells were seen in the hippocampus of P21 rats in the formalin group (Additional file 3: Figure S2C-S2F). Western blotting verified the significant reduction of NK-1R in the hippocampal tissue of the formalin group (Additional file 3: Figure S2G and S2H).

### Peripheral inflammatory pain-induced microglia increase/activation in the brain

**Fig. 2:**
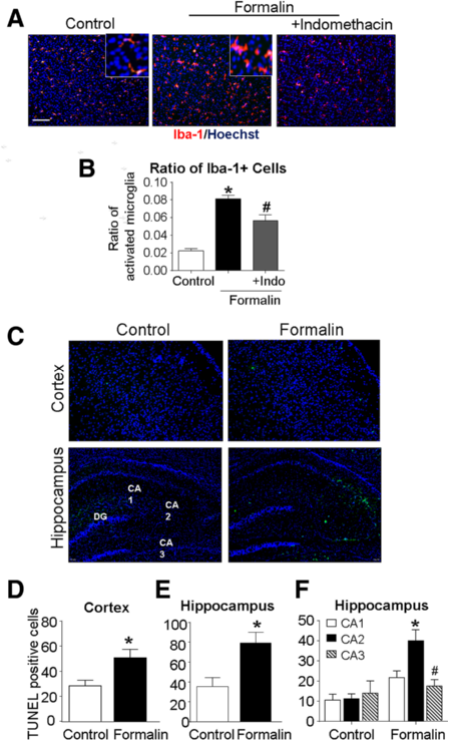
Neonatal peripheral inflammatory pain-induced microglia activation and neuronal cell death in the cortex and hippocampus. Microglia cells and neuronal cell death were measured using Iba-1 and TUNEL staining, respectively, in the cortex and hippocampus. **a**, **b** Immunohistochemical staining was performed 1 day after the last formalin injection. Iba-1-positive cells (red) were identified as microglia cells in the cortex. Nuclei were stained with Hoechst 33342 (*blue*). *Different shapes* of Iba-1-positive microglia cells at resting state (control) and activation state (formalin) are shown in the *insets* of enlarged magnification. *Scale bars* = 100 μM. **b** The ratio of Iba-1+ cells against total Hoechst + cells. Formalin injections significantly increased activated microglia while indomethacin (Indo) attenuated this event. *P < 0.05 vs. control, ^#^ *P* < 0.05 vs. formalin, ANOVA plus Bonferroni’s correction; *n* = 6–8 per group. **c** TUNEL staining (*green*) and Hoechst 33342 (*blue*) were used to evaluate cell death in the cortex and hippocampus. *Arrows* point to some TUNEL-positive green color cells. *Scale bars* = 100 μm. **d**, **e** Quantified data of TUNEL-positive cells/field in the cortex (**d**) and hippocampus (**e**). *P < 0.05 vs. control; *n* = 6–7 per group. **f** TUNEL-positive cells/field in the hippocampus in saline control and formalin groups. A marked increase in TUNEL-positive cells was seen in CA2. *P < 0.05 vs. CA1 or CA3, ANOVA plus Bonferroni’s correction; *n* = 6–7 per group

To further determine whether peripheral inflammatory pain could induce inflammatory responses in the brain, we measured microglia cells stained with ionized calcium-binding adaptor molecule-1 (Iba-1) in the cortex 1 day after the 3-day formalin insult. The number of Iba-1-positive cells in the cortex from the formalin group significantly increased comparing to that in control rats (Fig. 2a, b). Indomethacin co-applied with formalin significantly attenuated the microglia increase (Fig. 2b). Morphological assessments revealed that control group microglia generally showed ramified shape while most Iba-1-positive cells in the formalin group exhibited hypertrophied and bushy shapes consistent with activated microglial cells (Fig. 2a).

### Peripheral inflammatory pain-induced cortical and hippocampal cell death

One day after the 3-day formalin insult, TUNEL staining was performed in brain sections for the inspection of cell death. Spontaneous apoptotic cell death normally occurs in the developing brain [29]. The inflammatory insult significantly increased the number of TUNEL-positive cells in the cortex and hippocampus (Fig. 2c–f). This is consistent with our previous observation using the neuron-specific marker NeuN that formalin-induced neuronal cell death in the cortex, hippocampus, and hypothalamus [22]. Focusing on the hippocampus, we noticed that the hippocampal CA2 region had significantly more TUNEL-positive cells than that in the hippocampal CA1 and CA3 regions (Fig. 2f). The selective cell death of CA2 may be significant as damage to this region has recently been implicated in the loss of social memory [26].

### Inflammatory pain in neonates impaired axonal integrity and myelination in the juvenile brain

**Fig. 3:**
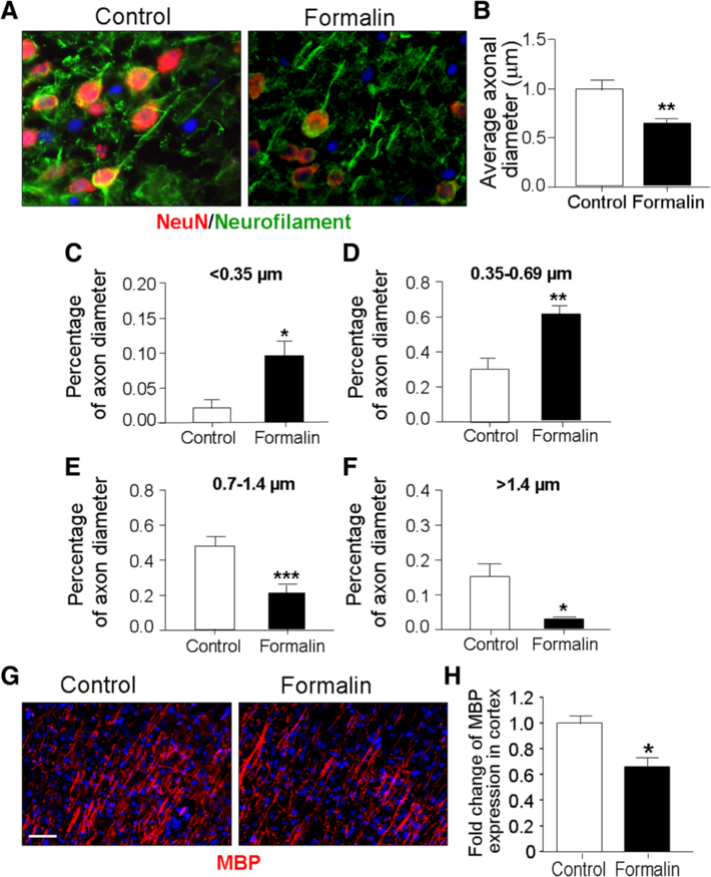
Neonatal inflammatory pain affected axonal development in the juvenile brain. Immunohistochemical staining was performed to determine whether peripheral inflammatory pain in neonates could affect axonal growth and myelination at P21. **a** Double-labeled images of NeuN (*red*) and neurofilament (NF; *green*) in the cortex of the control or formalin group. Nuclei were stained with Hoechst 33342 (*blue*). *Scale bars* = 20 μm. **b**–**f** Axon diameter was estimated by measuring the distance perpendicular to the center of the maximum diameter of the axon profile. Measured axons were categorized as small (<0.35 μm), medium (0.35–0.69 μm), large (0.7–1.4 μm), and extra-large (>1.4 μm) groups. *P < 0.05 vs. control; **P < 0.01 vs. control; ***P < 0.001 vs. control; *n* = 6 per group. **g** Images of MBP (*red*) in the cortex. Nuclei were stained with Hoechst 33342 (*blue*). *Scale bars* = 20 μm. **h** Juvenile rats subjected to neonatal inflammatory pain showed a significant decrease of MBP expression compared to control rats. *P < 0.05 vs. control; *n* = 6 per group

Axonal diameter may reflect its structural integrity and directly affects conductivity and electrophysiological properties. Prior to this study, it was unknown whether peripheral pain might have an impact on axonal integrity in the developing brain. In the control brain of P21 rats, axon diameter varied from 0.1 to 3 μm. The average value was 0.957 ± 0.079 μm with most of the axons being the medium (0.35–0.69 μm) or large (0.7–1.4 μm) size (Fig. 3a–f). Rats in the formalin group showed an average axonal diameter of 0.642 ± 0.027 μm (Fig. 3b), which was significantly smaller than the control axons (*n* = 6 assays, *P* < 0.01). There was a larger proportion of small- and medium-sized axons in the brain of formalin-treated rats (Fig. 3c–f) compared to the large-sized axons in controls. In the assessment of myelination of axon fibers, P21 rats subjected to neonatal inflammatory pain showed a significant reduction of myelin basic protein (MBP) in the IV/V cortical columns (Fig. 3g, h).

### Inflammatory pain in neonates diminished regenerative activities in the juvenile brain

**Fig. 4:**
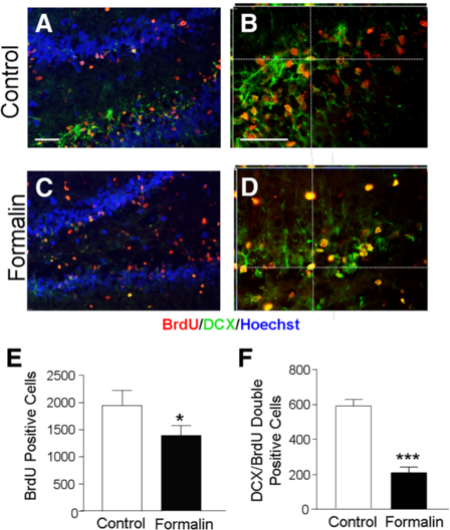
Neonatal inflammatory pain decreased neurogenesis in the juvenile brain. Immunofluorescent double labeling for DCX and BrdU was performed to examine neurogenesis in P21 rats. **a**–**d** Double-labeled images of BrdU (*red*) and DCX (*green*) in the dentate gyrus of the control (**a**, **b**) or formalin (**c**, **d**) group. Nuclei were stained with Hoechst 33342 (*blue*). **b**, **d** 3-D confocal images of BrdU (*red*) and DCX (*green*) in the dentate gyrus of the control and formalin groups. *Scale bars* = 50 μm. **e**, **f** Summarized data of the total numbers of DCX-positive and DCX/BrdU-positive cells in three sections of each animal. There were fewer BrdU-positive (**e**) and DCX/BrdU-positive cells (**f**) in the formalin group compared to the control group. ***P < 0.001 vs. control; *n* = 6 per group

To label newly formed cells in the developing brain, bromodeoxyuridine (BrdU; 5 mg/kg/day), a cell-proliferation marker, was injected intraperitoneally daily from P3 to P21. Double labeling of BrdU and doublecortin (DCX), a marker for neuronal precursor cells and immature neurons, was examined in the neurogenic subgranular zone of the hippocampus of P21 rats (Fig. 4). The number of BrdU-positive cells in the dentate gyrus of the formalin group was fewer than that of control group (Fig. 4e). Specifically, the number of newly formed neuronal cells (DCX+/BrdU+) in the dentate gyrus of the formalin group was significantly suppressed (Fig. 4f).

### Inflammatory pain in neonates altered expressions of ASD-related genes in the cortex of the juvenile brain

**Fig. 5:**
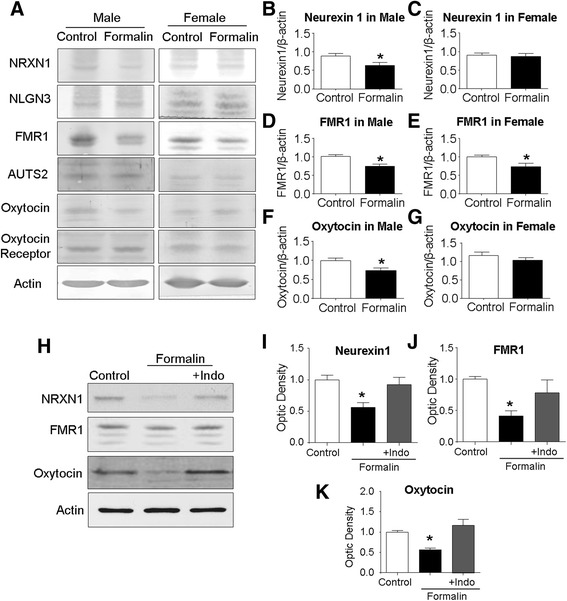
Neonatal peripheral inflammatory pain altered ASD-related genes in juvenile rats. Western blotting analysis was performed to determine the expression of ASD-related genes in the cortex of P21 rats subjected to saline and formalin injections at neonatal stage. **a** Representative Western blot bands of NRXN1, NLGN3, FMR1, AUTS2, oxytocin, and oxytocin receptor of male and female rats. **b**, **d**, and **f** Optic density of NRXN1 (**b**), FMR1 (**d**), and oxytocin (**f**) in the cortex of male rats. *P < 0.05 vs. control; *n* = 12–14 per group. **c**, **e**, and **g** Optic density of NRXN1 (**c**), FMR1 (**e**), and oxytocin (**g**) in the cortex of female rats. *P < 0.05 vs. control; *n* = 12–14 per group. **h**–**k** The expression of NRXN1, FMR1, and oxytocin in control and formalin groups of male sex (P21). The reductions induced by the formalin insult were all blocked by the anti-inflammatory treatment of indomethacin (Indo) co-applied with formalin. *P < 0.05 vs. control, ^#^ *P* < 0.05 vs. formalin group, ANOVA plus Bonferroni’s correction; *n* = 4 per group

ASD-associated gene expression was inspected in the cortical tissue of P21 juvenile rats subjected to either early formalin insults or saline injections. Western blotting was applied to measure expression of some key genes associated with ASD, including neurexin 1 (NRXN1), fragile X mental retardation 1 (FMR1), neuroligin3 (NLGN3), autism susceptibility gene 2 (AUTS2), oxytocin, and oxytocin receptor. The expression levels of neurexin 1, FMR1, and oxytocin all decreased in the cortex of male rats of the formalin group (Fig. 5a, b, d, and f). On the other hand, only the FMR1 expression was significantly reduced in the cortex of female rats that received formalin injections (Fig. 5a, c, e, and g). Indomethacin co-applied with formalin evidently blocked alterations of ADS-related genes in P21 male rats (Fig. 5h–k). No significant changes were seen in expressions of NLGN3, AUTS2, and oxytocin receptor in the cortex of either sex (data not shown).

### Inflammatory pain in neonates altered the expression of oxytocin receptor in the hippocampus of the juvenile brain

**Fig. 6:**
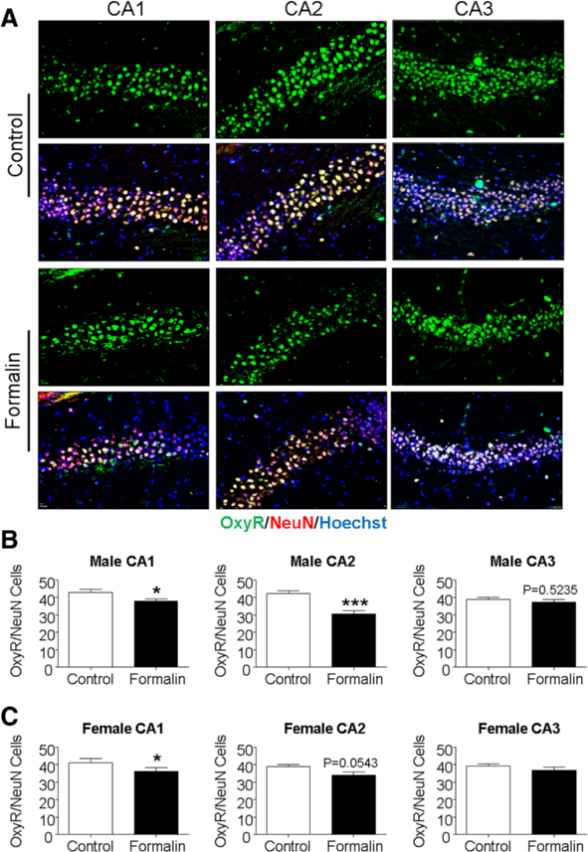
Neonatal peripheral inflammatory pain diminished the oxytocin receptor expression in the hippocampus of the juvenile brain. Immunohistochemical staining measured the expression and distribution of the oxytocin receptor in the CA1, CA2, and CA3 regions of the hippocampus. P21 rats were subjected to formalin injections or saline injections at P3–P5 age. **a** Oxytocin receptor-positive cells (*green*) and NeuN-positive cells (*red*, shown as *purple* due to overlay with *blue* of Hoechst 33342 nuclei staining) in the hippocampus from the control and formalin groups. The *first row* in each group shows oxytocin receptor only, and the *next row* shows the overlay images of all three immunostainings. *Scale bars* = 50 μM. **b**, **c** Quantified data from experiments in **a**. The numbers of oxytocin receptor/NeuN double-positive cells per survey area under × 20 magnification. These double-labeled cells in both CA1 and CA2 were significantly less in male rats in the formalin group comparing to male controls (**b**). A trend of reduced oxytocin receptor level was also seen in CA3. In female rats of the formalin group, a significant reduction of oxytocin-positive neurons was only seen in the CA1 region, although there was a trend of reduction in CA2 (**c**). *P < 0.05 vs. control, ***P < 0.001 vs. control; *n* = 6–7 per group

Recent studies suggest that the hippocampal CA2 region and the level of oxytocin receptor in this particular region are strongly related to social memory functionality [26, 30, 31]. Immunohistochemical analysis of oxytocin receptors was then focused on the hippocampus of P21 rats. It was seen that the oxytocin receptor was mostly colocalized with NeuN-positive cells (Fig. 6a). Significantly fewer oxytocin receptor/NeuN double-positive cells were detected in CA1 and CA2 regions of male and female rats in the formalin group (Fig. 6b, c). However, the downregulation of oxytocin receptor was more evident in male rats comparing to the marginal reduction in female rats (Fig. 6b, c).

### Inflammatory pain in neonates caused functional/behavioral deficits in juvenile rats

**Fig. 7:**
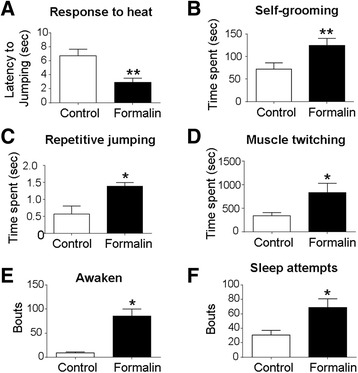
Neonatal inflammatory pain caused increased pain sensation, self-repetitive behaviors, and sleep disorder in juvenile rats. Hot-plate and repetitive behavior tests were performed to examine pain sensation and ASD-related activities in P21 juvenile rats. **a** In the hot-plate test, rats were placed individually on a hot plate of 55 °C. The latency time to jump was measured. Rats in the formalin group were more sensitive to the hot stimulation, showing a reduced latency to jump compared to controls. **P < 0.01 vs. control; *n* = 9–11 per group. **b**–**f** Six-hour monitoring of the HomeCage system for other repetitive behaviors and sleep pattern. HomeCage monitoring system monitored behavioral patterns of control and formalin-injected rats in a natural environment without human intervention. Rats in the formalin group showed significantly increases in repetitive grooming (**b**), spontaneous jumping (**c**), and muscle twitching (**d**) behaviors at P21. These animals showed increased frequencies of getting awake and trying to sleep compared to control rats during the same monitoring time (**e**, **f**). *P < 0.05 vs. control, **P < 0.01 vs. control; *n* = 16 per group

We examined the possibility that the acute inflammatory pain occurring in neonates could alter pain sensation later in juvenile rats. In the hot-plate test, P21 rats in the formalin group were much more sensitive to the thermal stimulus, showing significantly reduced latency in response to heat stimulus (Fig. 7a). In more behavioral surveillance using a HomeCage monitoring system in rat’s natural environment without human intervention, P21 rats in the formalin group spent significantly more time engaging in repetitive behaviors such as self-grooming, repetitive jumping, and spontaneous muscle twitching (Fig. 7b–d). These animals also showed sleeping disorder, exhibited as marked increases in bouts of awake and sleep activities (Fig. 7e, f). Additionally, rats in the formalin group displayed changes in olfaction-related activities and anxious behaviors (Additional file 4: Table S2 and Additional file 5: Table S3).

### Effects of neonatal inflammatory pain on spatial and social memory in juvenile rats

The Morris water maze test was performed to evaluate spatial memory in P21 rats [25]. During a 6-day training period starting from P15, the latency and distance travelled to the underwater platform improved similarly in both control and formalin groups (Additional file 6: Figure S3A and S3B). On day 7, all rats spent similarly more time in the target quadrant than in other quadrants (Additional file 6: Figure S3C and S3D). Since we detected significant changes in olfactory-related activities in P21 rats of the formalin group (Additional file 4: Table S2), the social transmission test of food preference was utilized to explore olfactory memory differences. This test, nevertheless, revealed no significant difference between saline and formalin groups (Additional file 6: Figure S3E and S3F).

In a social direct interaction test [26] of two trials with unfamiliar and familiar rats, respectively, normal juvenile rats spent shorter time in trial 2 with the familiar rat. However, no such time reduction was seen with male or female rats in the formalin group, indicating a deficit in social memory (Additional file 7: Figure S4A). This functional deficit was then verified with another two trial tests using two unfamiliar rats in both trials. Compared with normal controls, male rats in the formalin group showed less interest in unfamiliar rats while female rats did not exhibit significant deference from normal controls (Additional file 7: Figure S4B). In a five-trial social memory test with repeated exposures to the same familiar rats (trials 2 to 4), male rats but not female rats in the formalin group exhibited a social memory deficit in trial 3 (Additional file 7: Figure S4C).

### Inflammatory pain in neonates caused impaired social activity in juvenile rats

**Fig. 8:**
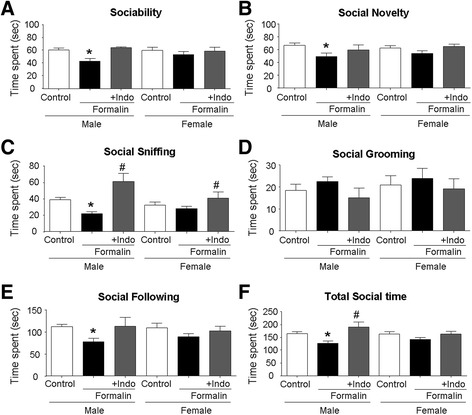
Neonatal inflammatory pain-induced delayed impairments in social activities and the blocking effects of the anti-inflammatory treatment. Social behavioral tests were carried out to evaluate long-term adverse impacts of the early inflammatory pain on social interactions in juvenile rats. The indomethacin (Indo) treatment was applied to delineate whether inflammation was a crucial mediator in the social behavior changes. **a** In the three chamber test, male rats, not female rats, in the formalin group, spent less time with the novel stranger and preferred to hide in an empty chamber compared to the control rats. Indomethacin (Indo) co-applied with formalin completely blocked the development of this abnormal behavior. *P < 0.05 vs. control; *n* = 16 in control and formalin group. *n* = 5 in indomethacin group. **b** In the social novelty test, since male rats in the formalin group preferred to stay with a familiar rat, their time with a novel stranger was significantly less than the time spent by controls. The indomethacin (Indo) treatment blocked the social abnormality. *P < 0.05 vs. control; *n* = 16 in control and formalin group. *n* = 5 in indomethacin group. **c**–**e** In the social interaction test, male rats in the formalin group showed reduced time in social sniffing and social following, although no change was seen with social grooming. Indomethacin (Indo) reversed the behavioral changes. Female rats which received formalin did not show significant deficits in these tests. *P < 0.05 vs. control, ^#^ *P* < 0.05 vs. formalin; *n* = 16 in control and formalin group. *n* = 5 in indomethacin group. **f** A summarized data analysis of all social behavioral tests for male and female rats. The total social time was significantly less with male rats in the formalin group, and the phenotype was prevented by the anti-inflammation treatment. Again, female rats were much more resistant to the formalin insult, showing no social behavior deficits at the juvenile age. *P < 0.05 vs. control, ^#^ *P* < 0.05 vs. formalin; *n* = 16 in control and formalin group. *n* = 5 in indomethacin group. ANOVA plus Bonferroni’s analysis was applied to all comparisons in this experiment

In addition to increased repetitive behaviors, impaired social activities and difficulties in social communication are some clinical syndromes of ASD [7]. In a three-chamber test of P21 rats, male but not female rats in the formalin group showed a significant preference to stay in the empty chamber. They also preferred to stay in the chamber with familiar rats in the social novelty test. As a result, these male rats spent significantly less time in social interactions (Fig. 8a, b). Other social interaction tests showed similar reductions in social activities including social sniffing and social following (Fig. 8c, e). Though there was no difference in social grooming between formalin and control animals, the total social time for all tested social activities was significantly less for male rats in the formalin group (Fig. 8a–f). In contrast, the social activity deficits were not observed with female rats in the formalin group (Fig. 8). These results verified that male rats with early inflammatory pain experience were more vulnerable to developing abnormal social behaviors than female rats. These results support the idea that inflammatory pain and increased inflammatory factors are critical mediators in the development of ASD-like behaviors. All detected social behavioral deficits were essentially prevented by the co-applied anti-inflammation pain treatment indomethacin (Fig. 8a–f).

### Comparisons of the symptoms in the animal model and human cases

To validate whether the observation on the inflammatory pain-induced pathological, pathophysiological, and psychiatric changes are consistent with the symptoms in ASD patients, we compared our results to published clinical data in order to better understand the clinical significance of this investigation (Table 1). As shown in Table 1, there are overwhelmingly similarities between the pathological process, cellular/molecular alterations, and functional/behavioral phenotypes between the animal study and clinical observations.

**Table 1:** Comparison between the formalin-induced inflammatory pain model and clinical observations of ASD patients

Animals model	Clinical data of ASD patients	Consistency
Increases of inflammatory cytokines such as TNF-α and IL-1β in the blood and the brain	Elevation of TNF-α in cerebrospinal fluid of autistic children [66].	Yes
	Elevated levels of the pro-inflammatory cytokine, including IL-1, IL-6, IL-12, IL-23, TNF-α, and BDNF [67].	
	A trend toward a significantly increased production of IL-6 and TNF-α in whole blood [68].	
	Increased plasma concentrations of IL-1b, IL-1RA, IL-5, IL-8, IL-12(p70), IL-13, IL-17, and GRO-a [69].	
Impaired neurogenesis	Neuropathological developmental changes in the brain reflect multiregional dysregulation of neurogenesis, neuronal migration, and maturation [70].	Yes
Dysregulation of neurexin-1	Two putative missense structural variants were identified in the neurexin 1β gene [71].	Possibly yes
Disruption of oxytocin system and therapeutic effect of oxytocin	Significantly lower plasma oxytocin levels [72].	Yes
	A significant correction of repetitive behaviors following oxytocin infusion [73].	
Self-grooming and repetitive jumping (repetitive behaviors)	Significantly higher frequency and longer duration of repetitive and stereotyped behaviors [74].	Yes
	Ritualistic and stereotypical behavior [75].	
Sleep problem	Insomnia associated with neurochemical (abnormalities in serotonergic transmission or melatonin levels), psychiatric (anxiety), and behavioral (poor sleep habits) etiological factors [76, 77].	Yes
	Treatments for insomnia show promise for behavioral/educational interventions [78].	
Reduction of FMRP	Significantly reduced levels of FMRP protein [79].	Yes
Social memory deficits	Impaired on immediate and delayed recall of faces and of family scenes and impaired spatial working memory. Defective integrity of verbal working memory and impaired spatial working memory [80].	Yes
Axonal impairments	Area-specific changes below anterior cingulate cortex (ACC) included a decrease in the largest axons that communicate over long distances. Overexpression of the growth-associated protein 43 kDa accompanied by excessive number of thin axons that link neighboring areas. In the orbitofrontal cortex (OFC), axons had decreased myelin thickness [24].	Yes or partially yes
	Decreased levels of proteins associated with myelination and increased synaptic and energy-related proteins in the prefrontal cortex. Opposite directional changes were found for myelination and synaptic proteins in the cerebellum [81].	
Deficits in social activities	Developmental delays in social interaction, language, and imaginative function [82]	Yes or partially yes
Altered oxytocin system in CA2 region	Oxytocin is a key factor in CA2 regional social memory function [26, 83]. No direct human data available.	Potentially yes
ASD phenotypes are more prominent in male than in female animals.	Sexually dimorphic responses to early life stress are linked to two developmental disorders: affective problems (greater female prevalence) and ASD (greater male prevalence) [84, 85].	Yes,
Abnormal cell death	The abnormal apoptosis found in autism from postmortem [86].	Yes

## Discussion

The present investigation provides novel and comprehensive cellular, molecular, and behavioral evidence that acute but relatively severe neonatal inflammatory pain can trigger lasting systemic inflammatory responses and pathological alterations that may generate a vulnerable environment for the development of ASD-like syndrome. Formalin-induced inflammatory pain increases major inflammatory factors TNFα and IL-1β not only in the blood circulation but also in the brain. This upregulation of inflammatory factors persisted even after the disappearance of local tissue damage. Significant neuronal cell death in the cortex and hippocampus CA1/CA2 regions was observed after the peripheral inflammatory stimuli, accompanied by impaired neuronal axons and reduced neurogenesis. Moreover, the inflammatory pain led to long-term regulation of ASD-associated genes NRXN1, FMR1, and oxytocin/oxytocin receptor in the brain. These genetic alterations, increased repetitive behaviors, and deficient social memory/interactions all are analogous to patients with ASD and/or fragile X syndrome. Consistent with clinical cases, these pathological phenotypes are more prominent in males than in females. As a mechanistic verification and of clinical importance, these abnormal phenotypes can be largely prevented with an anti-inflammatory pain intervention using the clinical drug indomethacin.

Previous work reported that exposure to repetitive inflammatory pain during the development of high brain plasticity is associated with neuronal cell death, neuroinflammation, modulations in pain sensation, and adverse changes in brain structure and function [22, 32, 33]. Due to the plasticity of sensory connections in the neonatal period, early damage in infancy can cause prolonged structural and functional alterations in pain pathways that can last into later life stages [32, 33]. For example, inflammatory pain experienced during the postnatal period may cause abnormal adult behaviors such as increased anxiety, altered pain sensitivity, hyperactivity, self-destructive behavior, or reduced social behaviors [32, 33]. In clinical studies, premature neonates exposed to painful experiences are more likely to develop chronic abnormalities compared to full-term infants. For example, circumcised infants showed a stronger pain response to subsequent routine vaccination than uncircumcised infants [34]. On the other hand, there has been no research focusing on the relationship between neonatal inflammatory pain and the prevalence of ASD.

In the study of postmortem brain from ASD patients, elevated cytokines and chemokines and activated microglia were observed [35, 36]. A recent review pointed out that premature babies are more vulnerable to infections and inflammation that can lead to neurodevelopmental problems and higher risk for ASD [12]. In an animal study, maternal infections caused by multiple intraperitoneal injections of lipopolysaccharide damaged the layer formation of the fetal brain, possibly linked to neuropsychiatric disorders, such as schizophrenia and autism [37]. A clinical study with 1.2 million pregnancies showed that the risk of autism in the children of women with the highest levels of C-reactive protein, a well-known marker of inflammation, was 43 % higher than women with the lowest levels [38]. Another study provided new evidence that mothers who have autoimmune diseases associated with excess and/or chronic inflammation could be at increased risk of having children with ASD [39]. ASD patients frequently showed widespread inflammation as indicated by elevated inflammatory cytokines in both the brain and blood similar to those in autoimmune disease, signifying the importance of the inflammatory response on the development of ASD [40, 41]. Specifically, ASD patient’s peripheral blood cells secrete higher levels of TNF-α, IL-1β, and IL-6 [42, 43]. Our current findings are in line with these observations, showing systemic and lasting elevations of TNF-α, IL-1β, and activation of microglia in the brain. The link between inflammation and ASD pathology was strongly supported by the success of indomethacin in protecting against inflammatory pain-induced changes.

IL-1β-induced inflammation inhibited hippocampal neurogenesis [44]. Similarly, we observed reduced neurogenesis in the dentate gyrus of the formalin group, which may relate to the IL-1β increase in the brain. Conversely, one previous study in neonatal rats found increased hippocampal neurogenesis at P22 after a Freund’s adjuvant injection [45]. The contrasting result may be due to apparent differences in the inflammatory insults and in the timing and severity of the insult and measurements in a particular model. Inflammatory pain may result from the increased excitability of peripheral nociceptive sensory fibers activated by inflammatory mediators [46]. Alterations in pain pathways such as pain-related receptor expression can last into the adolescent or adult stage [16]. NK-1R, an activator of nociception-induced spinal central sensitization, is reduced in rat models of chronic pain and stress [28]. Consistent with these findings, rats exposed to early pain in our study had reduced NK-1R expression, which is likely an event related to enhanced pain sensitivity in adolescents/juvenile rats.

We show here in juvenile rats that early neonatal inflammatory pain causes important morphological alterations in the developing brain, including axonal damage and reduced MBP. These alterations resemble some changes caused by activated cytokine responses seen in prenatal stress [47]. A previous study on inflammatory lesions in multiple sclerosis patients revealed that axonal density changes may be caused by the release of inflammatory mediators [48]. It is likely that the persistent elevation of inflammatory factors in the brain is largely responsible for the axonal impairment. The significant axonal changes imply that altered neuronal transduction along these nerve fibers must take place after the inflammatory pain and chronic cytokine upregulation.

Repetitive and uncontrolled behaviors such as repetitive grooming, jumping, and muscle twitching are prominent features in human and animals with autism-like disorders [49, 50]. In our study, the time spent in twitching and doing repetitive jumping and grooming behaviors in juvenile rats were significantly increased in the formalin group. In general, rodents prefer social environments over solitary ones. They prefer to engage a novel partner rather than a familiar one. Strikingly, juvenile rats subjected to early inflammatory pain exhibit noticeable dysfunction in social interaction tests. In addition to social deficits, children with ASD appear to experience sleep problems more frequently than healthy children [7]. This is consistent with our observation that juvenile rats in the formalin group showed disrupted sleep behavior. Collectively, the functional and behavioral disorders in the animal model resemble the syndromes of ASD children.

Although memory loss has not been a diagnostic criterion for ASD, it is a common difficulty experienced by ASD patients [51]. In social memory tests, formalin-treated animals, especially male rats, exhibited an impaired social memory. Coincidently, we observed that formalin stimuli increased more TUNEL-positive cells in the hippocampal CA2 region. Previous research showed the social responsiveness is reduced in rodents with hippocampal lesions [52]. It is likely that the increased cortical and hippocampal neuronal cell death contribute, at least partly, to the development of abnormal social behaviors. Recent data revealed that the CA2 region is essential for social memory [26]. Our immunostaining data showed reduced oxytocin receptor in CA1 and CA2 regions of male rats in the formalin group. These findings raise the possibility that CA2 damages and abnormal gene expression induced by early inflammatory pain play a critical role in social memory impairment.

Many studies have been investigating the connection between genetic variation and ASD. Genome-wide association studies (GWAS) for ASD have identified few potential loci associated with ASDs [53]. NRXN1 was implicated as an autism susceptibility gene, though changes in this gene alone are not always detrimental [54]. For example, mice with a deletion of NRXN1 spend more time grooming but also show improved motor learning [54]. NRXN1 knockout mice display increased responsiveness and accelerated habituation to novel environments [55]. These data suggest that mutation or deletion of NRXN1 alone is not sufficient to cause ASD. In the inflammatory pain model, we detected significant decreases of NRXN1 and FMR1 expression in the cortex. Being a sub-category of ASD, fragile X syndrome is identified as a single gene inherited disorder due to mutations or deficiency of FMR1 [56]. FMR1 mutation or deletion has shown autism-like behaviors such as impaired social activity, anxiety, and reduced behavioral flexibility [56]. A wealth of studies has implicated oxytocin and the oxytocin receptor in the mediation of social behaviors and social memory, suggesting that failures in this system may be associated with ASD [57]. Decreased oxytocin and its receptor signal can result in low social activity and autism-like behaviors, and this change in oxytocin system is commonly detected in ASD patients [57]. Our study shows a significantly reduced oxytocin level in the cortex of formalin-treated rats. We did not detect significant changes in the expressions of NLGN3 or AUTS2 in formalin-treated rats. Alteration of NLGN3 contributes to the induction of autism-related behaviors [54, 58]. However, recent clinical investigations showed that NLGN3 may not be a major disease gene in ASD [54]. It is possible that although ASD is associated with multiple genes, the development of ASD does not require participation of all related genes.

Some important issues remain to be better addressed. Males are approximately four times more likely than females to be diagnosed with ASD [59, 60]. It has been hypothesized that prenatal sex steroids may affect fetal brain structure and function and consequently influences postnatal behavior [61]. Whether the high incidence of ASD in male can be explained by the levels of sex hormones in postnatal babies is obscure. Different levels of sex hormones are detected in fetal surroundings and human neonates [62]. As estrogen and progesterone have neuroprotective and anti-inflammatory effects, it might be possible that these sex hormones contribute to the low incidence of ASD in female [63, 64]. There are also several limitations in this investigation. Most children with ASD are at a normal gestational age at birth and are not treated by painful procedures. Whether inflammation pain shows similar pathological and etiological impacts on full term infants and adults remains to be examined. It is also to note that inflammation and pain are distinct insults although may sometimes reciprocal. The pathogenic effects of pain and inflammation may play distinctive roles in ASD, while this is unclear based on available data.

The inflammation insult tested in this investigation (two hindpaws formalin injections for three consecutive days) is relatively severe. In our preliminary tests, we found inflammatory reaction and pathological consequences to formalin-induced inflammatory pain depended on the severity of the insult. A single paw one-time formalin injection elicited mild changes sometimes without statistical significance. A clear demonstration of the “dose-dependent” pathogenesis for ASD development requires a future investigation. Considering that formalin can act as a pro-oxidative neurotoxicant, it may be necessary to verify the observations in this investigation using other inflammatory pain agents such as carrageenan, zymosan, or complete Freund’s adjuvant that are known to trigger longer inflammatory pain responses than formalin [65]. Based on our data, however, an irritating insult that triggers comparable pain and neuroinflammatory reactions should bear similar pathogenic consequence as shown with formalin. It will also be interesting to see whether the beneficial effects of anti-inflammation treatment may last longer beyond the juvenile age or continual treatments are needed for long-term effects.

## Conclusions

The cellular, molecular, and behavioral examinations in the inflammatory pain model of neonatal rats demonstrate significant alterations consistent with the pathological, pathophysiological, genetic, and psychological/psychiatric changes in ASD children. The higher incidence of ASD syndromes in male animals resembles the unique feature of clinical ASD cases. Importantly, an anti-inflammation treatment using indomethacin effectively prevents all ASD-like alterations. We propose that repeated inflammatory pain suffered by premature neonates is one of the important environmental risk factors leading to the development of ASD-like syndromes, while anti-inflammation and analgesic treatments should be explored as a prevention therapy before the development of ASD.

### Ethics approval and consent to participate

All animal experiments in this investigation were approved by the Institutional Animal Care and Use Committee (IACUC) at Emory University and met with NIH guidelines for animal uses.

### Consent for publication

Not applicable.

### Availability of data and materials

Information about the animal model, experimental methods and data described in this paper are available to scientific and medical communities for review, verification, and research studies.

## Additional files

Additional file 1: Figure S1. Effects of neonatal peripheral inflammatory pain on paw edema, locomotion, and brain inflammation. The inflammatory pain animal model was generated by S.C. injection of 5 % formalin (5 μl) into the hindpaws of P3–P5 rat pups. A. Representative photographs of rat’s right hindpaw 3 and 11 days after saline and formalin injections. B. Rats in the formalin group developed hindpaws edema after the first injection (P3). The edema reached peak in P8 rats (5 days after the first injection). The local edema completely subsided after P16. N = 12 per group. C and D. Travelled distance and velocity were measured using the TopScan System at P10, P15, and P20 rats. At P10 and P15, the formalin group showed significantly less travelled distance and lower velocity than the control group. The differences were absent among P20 rats. * *P* < 0.05 vs. control; n = 6-7 per group. (TIF 1699 kb) Additional file 2: Table S1. Body weight in male and female rats received saline control or formalin insult. (DOC 39 kb) Additional file 3: Figure S2. Neonatal inflammatory pain decreased the substance P and NK-1R expressions in the brain. qRT-PCR and immunohistochemical analyses were performed to examine the effect of neonatal peripheral inflammatory pain on substance P and NK-1R expressions in hippocampal of P6 and/or P21 rats. A and B. In the qRT-PCR analysis, substance P was not changed in both P6 male and female rats (A). In the P21 male rats with formalin insult, substance P was significantly reduced, whereas this alteration was disappeared in indomethacin treatment (B). C-F. In immunohistochemistry analysis, cells were visualized with Hoechst 33342 (blue) and those with substance P receptors were labeled with anti-substance P receptor antibody (NK-1R; green) in the hippocampal region of the control or formalin group. Scale bars = 50 μm. In E and F, quantified bar graphs show reduced NK-1R by formalin. ** *P* < 0.01 vs. control; *** *P* < 0.001 vs. control, ANOVA plus Bonferroni's correction; n = 6 per group. G and H. Western blotting confirmed a significant reduction of NK-1R expression in the formalin group. * *P* < 0.05 vs. control, ANOVA plus Bonferroni's correction; n = 6 per group. (TIF 4583 kb) Additional file 4: Table S2. Bouts of home cage behavior. (DOC 69 kb) Additional file 5: Table S3. Time spent in home cage behavior (min). (DOC 49 kb) Additional file 6: Figure S3. Neonatal inflammatory pain didn’t cause spatial and olfactory memory impairments in juvenile rats. A-D. Juvenile male and female rats in the control or formalin groups were evaluated for spatial memory in the Morris water maze test. A. Latency to escape to the platform during the 6 day training session. There were no significant differences between the groups. n = 13 per group. B. Swim distance to reach the platform during training. There were not significant differences between groups. n = 13 per group. C and D. The place preference test was conducted at day 7 when the platform was removed after the last training day. All animals spent more time in the target quadrant (platform quadrant) (C), but there were no statistical differences between groups (D). n = 13 per group. E and F. Juvenile male and female rats in control or formalin groups were evaluated for olfactory memory in the social transmission of food preference test. There were no significant differences in the amount of total food consumed and the percentage of preference for cued food (almond) between groups. n = 8-15 per group. (TIF 1085 kb) Additional file 7: Figure S4. Neonatal peripheral inflammatory pain caused social memory impairment in male juvenile rats. Direct interaction and five-trial social memory tests were performed to clearly reveal the effect of neonatal peripheral inflammatory pain on social memory. A and B. Direct interaction test using the same (A) or different (B) rats in the two trials. A. Both male and female rats in the formalin group displayed increased exploring time (unchanged sociability), but the control group showed decreased exploring time (decreased sociability). * *P* < 0.05 vs. control; n = 6-7 per group. B. When subject rats were exposed to a novel rat, both groups explored new stimulus rats similarly. * *P* < 0.05 vs. control; n = 6-7 per group. C. Five-trial social memory assay. Male, but not female, rats in the formalin group dishabituated to the same rat during four trials and habituated to a novel rat (trial 5). * *P* < 0.05 vs. control; n = 6-7 per group. (TIF 871 kb)

## Abbreviations

ANOVA: analysis of variance
APA: American Psychiatric Association
ASD: autism spectrum disorder
AUTS2: anti-autism susceptibility gene 2
AUTS2: autism susceptibility gene 2
BrdU: bromodeoxyuridine
DCX: doublecortin
FMR1: fragile X mental retardation 1
IACUC: institutional animal care and use committee
Iba-1: ionized calcium binding adaptor molecule-1
IL-1β: interleukin-1 beta
MBP: myelin basic protein
NeuN: neuronal nuclei
NICU: neonatal intensive care unit
NK-1R: neurokinin 1 receptor
NRXN1: neurexin 1
OCT: optimal cutting temperature
P3: postnatal day 3
PBS: phosphate buffered saline
PDD-NOS: pervasive developmental disorder
qRT-PCR: quantitative real-time polymerase chain reaction
sc: subcutaneous injection
SEM: standard error of the mean
TBST: tris-buffered saline with Tween-20
TNF-α: tumor necrosis factor alpha
TUNEL: terminal deoxynucleotidyl transferase dUTP nick end labeling
