# Supplementary figures and images for: Neonatal inflammatory pain and systemic inflammatory responses as possible environmental factors in the development of autism spectrum disorder of juvenile rats
Source: J Neuroinflammation. 2016 May 16;13:109. doi: 10.1186/s12974-016-0575-x (PMC4867541; doi:10.1186/s12974-016-0575-x)

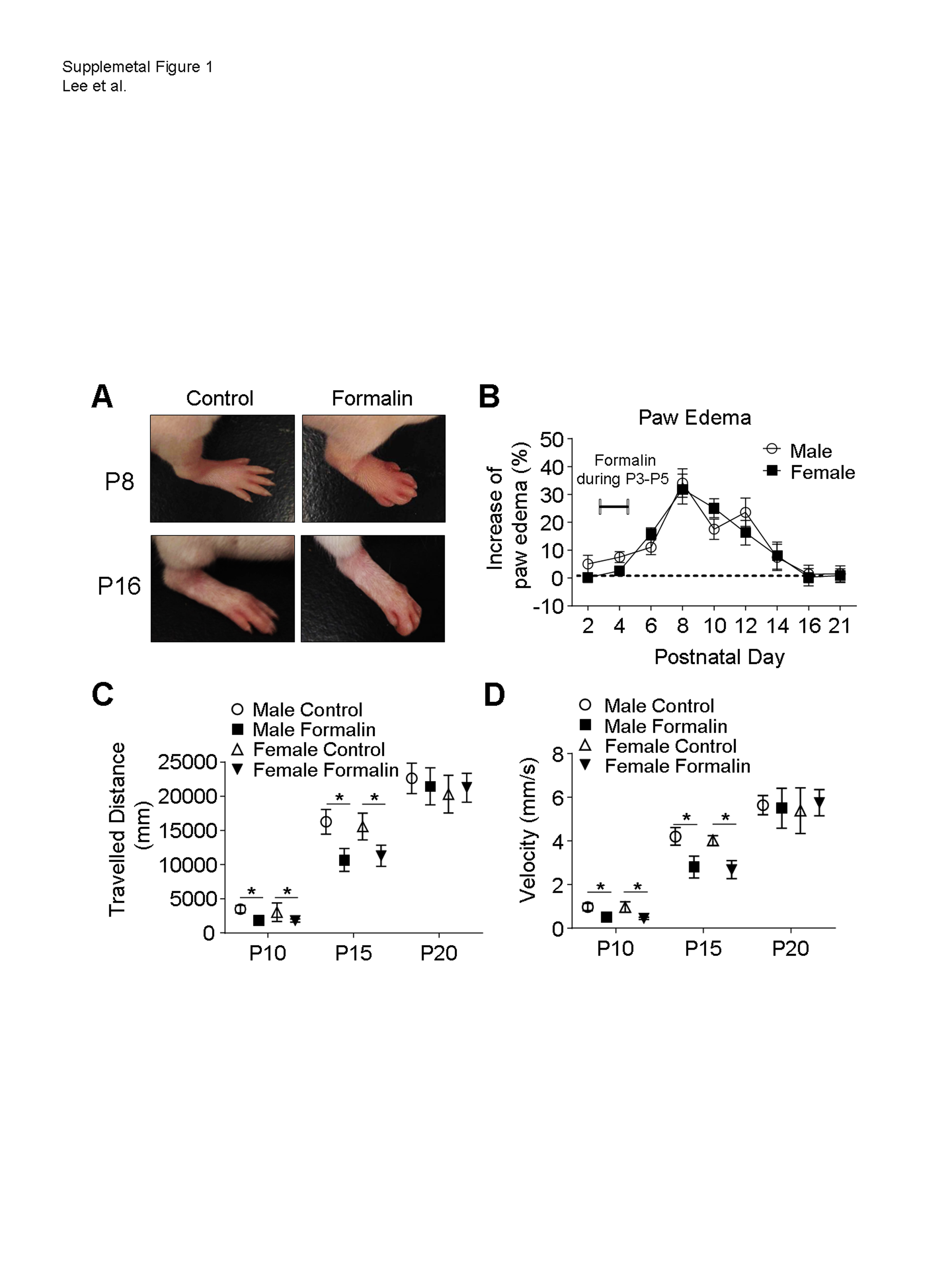

Supplement: Additional file 1: Figure S1. — Effects of neonatal peripheral inflammatory pain on paw edema, locomotion, and brain inflammation. The inflammatory pain animal model was generated by S.C. injection of 5 % formalin (5 μl) into the hindpaws of P3–P5 rat pups. A. Representative photographs of rat’s right hindpaw 3 and 11 days after saline and formalin injections. B. Rats in the formalin group developed hindpaws edema after the first injection (P3). The edema reached peak in P8 rats (5 days after the first injection). The local edema completely subsided after P16. N = 12 per group. C and D. Travelled distance and velocity were measured using the TopScan System at P10, P15, and P20 rats. At P10 and P15, the formalin group showed significantly less travelled distance and lower velocity than the control group. The differences were absent among P20 rats. * P < 0.05 vs. control; n = 6-7 per group. (TIF 1699 kb) [file 12974_2016_575_MOESM1_ESM.tif]

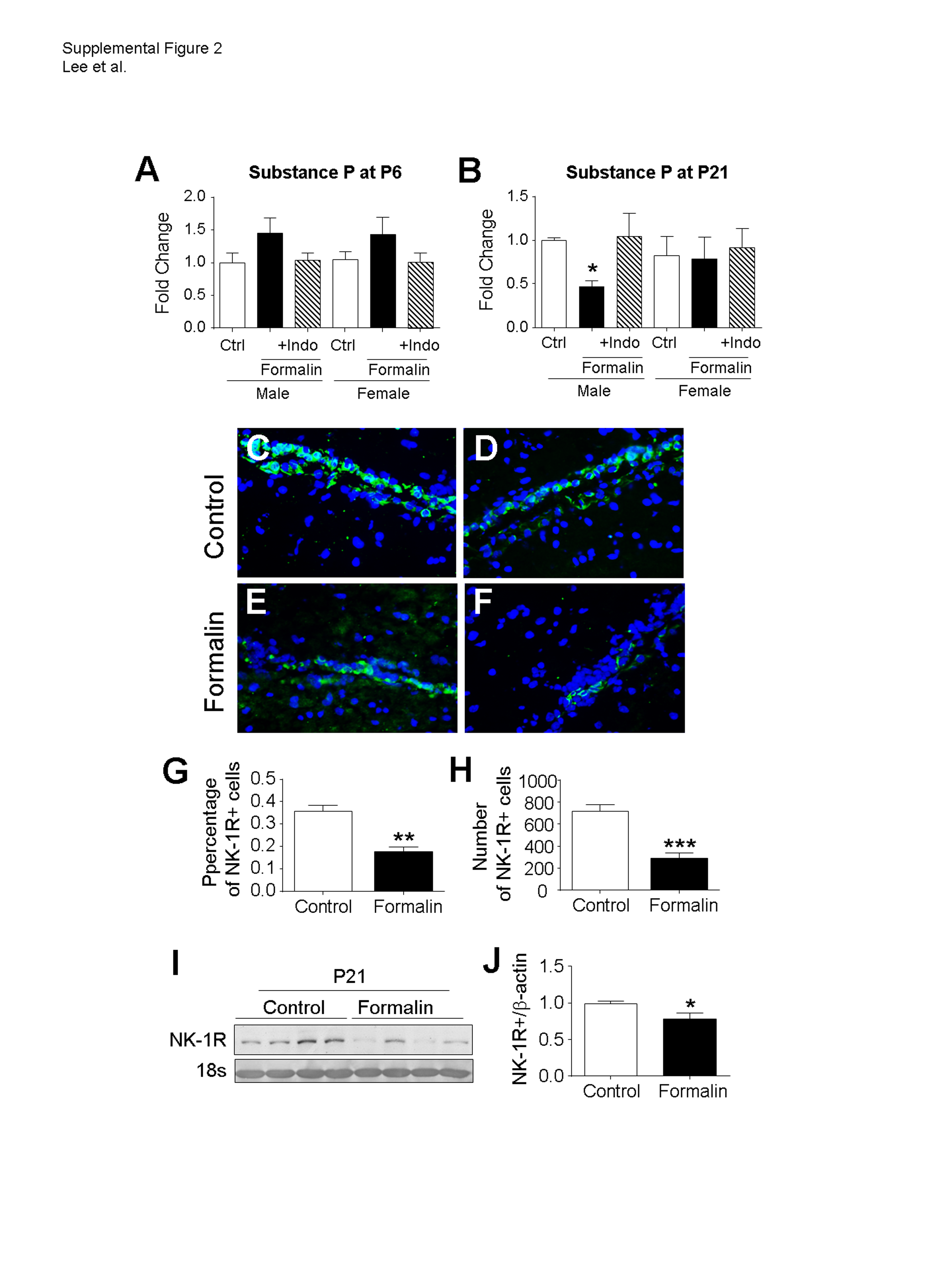

Supplement: Additional file 3: Figure S2. — Neonatal inflammatory pain decreased the substance P and NK-1R expressions in the brain. qRT-PCR and immunohistochemical analyses were performed to examine the effect of neonatal peripheral inflammatory pain on substance P and NK-1R expressions in hippocampal of P6 and/or P21 rats. A and B. In the qRT-PCR analysis, substance P was not changed in both P6 male and female rats (A). In the P21 male rats with formalin insult, substance P was significantly reduced, whereas this alteration was disappeared in indomethacin treatment (B). C-F. In immunohistochemistry analysis, cells were visualized with Hoechst 33342 (blue) and those with substance P receptors were labeled with anti-substance P receptor antibody (NK-1R; green) in the hippocampal region of the control or formalin group. Scale bars = 50 μm. In E and F, quantified bar graphs show reduced NK-1R by formalin. ** P < 0.01 vs. control; *** P < 0.001 vs. control, ANOVA plus Bonferroni's correction; n = 6 per group. G and H. Western blotting confirmed a significant reduction of NK-1R expression in the formalin group. * P < 0.05 vs. control, ANOVA plus Bonferroni's correction; n = 6 per group. (TIF 4583 kb) [file 12974_2016_575_MOESM3_ESM.tif]

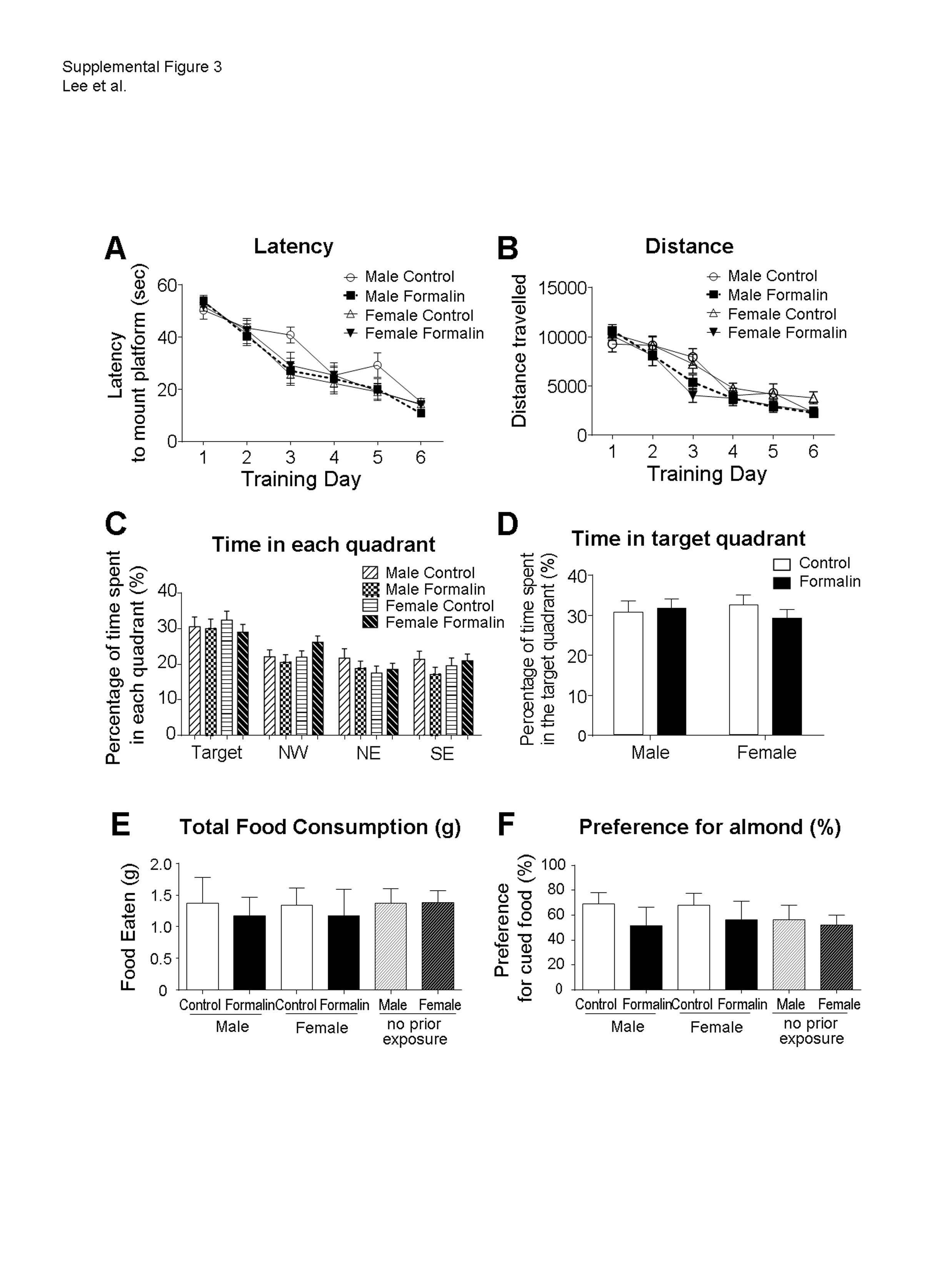

Supplement: Additional file 6: Figure S3. — Neonatal inflammatory pain didn’t cause spatial and olfactory memory impairments in juvenile rats. A-D. Juvenile male and female rats in the control or formalin groups were evaluated for spatial memory in the Morris water maze test. A. Latency to escape to the platform during the 6 day training session. There were no significant differences between the groups. n = 13 per group. B. Swim distance to reach the platform during training. There were not significant differences between groups. n = 13 per group. C and D. The place preference test was conducted at day 7 when the platform was removed after the last training day. All animals spent more time in the target quadrant (platform quadrant) (C), but there were no statistical differences between groups (D). n = 13 per group. E and F. Juvenile male and female rats in control or formalin groups were evaluated for olfactory memory in the social transmission of food preference test. There were no significant differences in the amount of total food consumed and the percentage of preference for cued food (almond) between groups. n = 8-15 per group. (TIF 1085 kb) [file 12974_2016_575_MOESM6_ESM.tif]

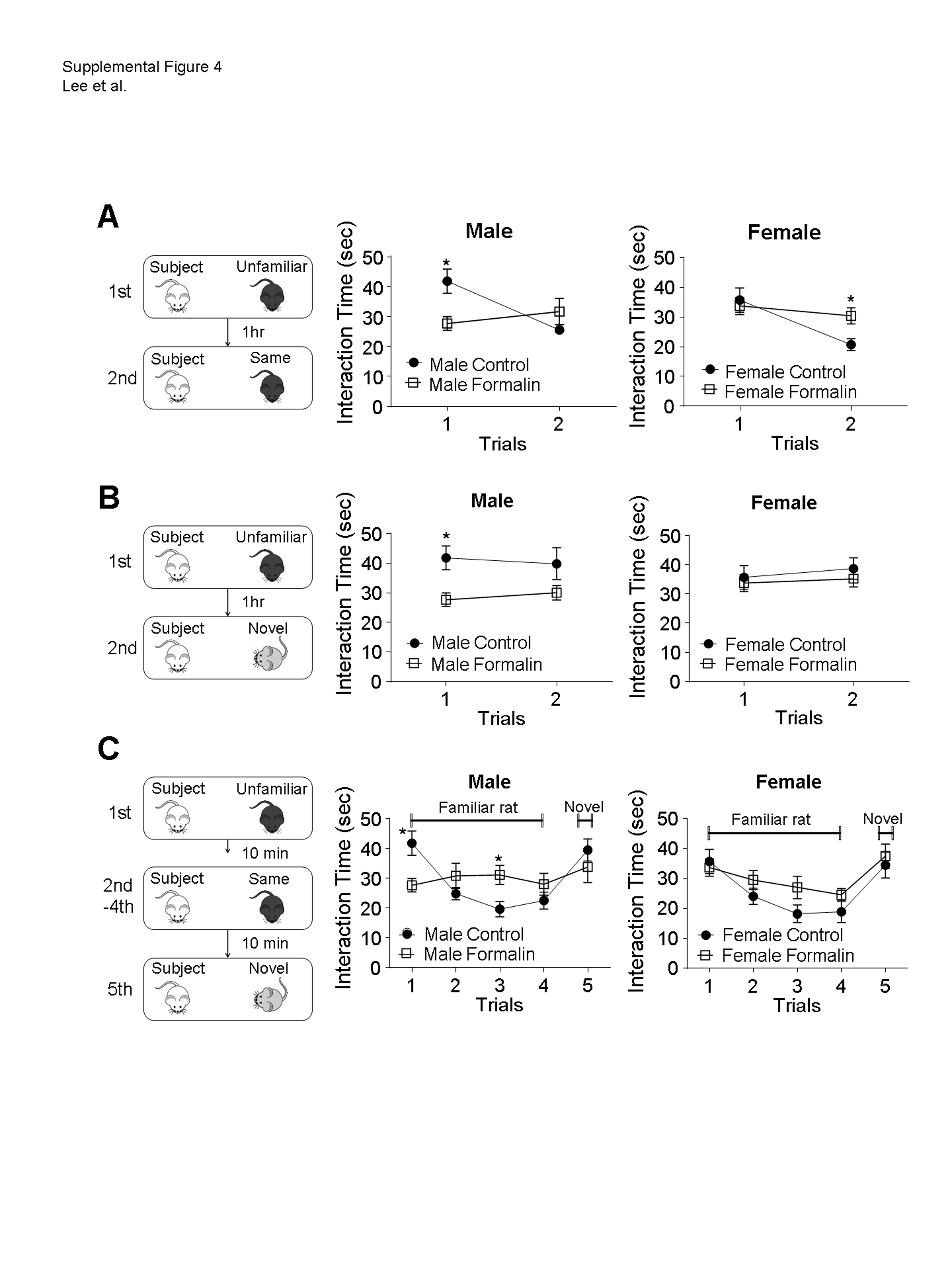

Supplement: Additional file 7: Figure S4. — Neonatal peripheral inflammatory pain caused social memory impairment in male juvenile rats. Direct interaction and five-trial social memory tests were performed to clearly reveal the effect of neonatal peripheral inflammatory pain on social memory. A and B. Direct interaction test using the same (A) or different (B) rats in the two trials. A. Both male and female rats in the formalin group displayed increased exploring time (unchanged sociability), but the control group showed decreased exploring time (decreased sociability). * P < 0.05 vs. control; n = 6-7 per group. B. When subject rats were exposed to a novel rat, both groups explored new stimulus rats similarly. * P < 0.05 vs. control; n = 6-7 per group. C. Five-trial social memory assay. Male, but not female, rats in the formalin group dishabituated to the same rat during four trials and habituated to a novel rat (trial 5). * P < 0.05 vs. control; n = 6-7 per group. (TIF 871 kb) [file 12974_2016_575_MOESM7_ESM.tif]
