# Supplementary material for: Neonatal inflammatory pain and systemic inflammatory responses as possible environmental factors in the development of autism spectrum disorder of juvenile rats
Source: J Neuroinflammation. 2016 May 16;13:109. doi: 10.1186/s12974-016-0575-x (PMC4867541; doi:10.1186/s12974-016-0575-x)
Supplement: Additional file 2: Table S1. — Body weight in male and female rats received saline control or formalin insult. (DOC 39 kb) [file 12974_2016_575_MOESM2_ESM.doc]

**Supplemental Table 1.** Body weight in male and female rats received saline control or formalin insult

| Days | Male Control (g)  (n=5) | | | Male Formalin (g)  (n=5) | | | Female Control (g)  (n=5) | | | Female Formalin (g)  (n=5) | | |
| --- | --- | --- | --- | --- | --- | --- | --- | --- | --- | --- | --- | --- |
| 0 | 4.45 | ± | 0.50 | 4.60 | ± | 0.26 | 4.13 | ± | 0.51 | 4.47 | ± | 0.30 |
| 3 | 11.90 | ± | 1.12 | 11.80 | ± | 0.39 | 10.17 | ± | 1.21 | 10.28 | ± | 0.56 |
| 5 | 15.47 | ± | 0.52 | 13.17 | ± | 1.20 | 15.23 | ± | 0.57 | 12.97 | ± | 1.21 |
| 7 | 18.42 | ± | 1.01 | 13.98 | ± | 1.45 | 17.80 | ± | 0.53 | 14.07 | ± | 1.15 |
| 9 | 25.88 | ± | 0.50 | 22.82 | ± | 1.67 | 25.57 | ± | 0.92 | 22.37 | ± | 1.83 |
| 12 | 35.25 | ± | 1.36 | 34.03 | ± | 2.50 | 33.12 | ± | 0.90 | 31.85 | ± | 1.96 |
| 15 | 46.88 | ± | 1.24 | 43.63 | ± | 1.27 | 44.68 | ± | 1.16 | 42.90 | ± | 1.47 |
| 18 | 56.45 | ± | 2.32 | 56.62 | ± | 1.62 | 54.48 | ± | 1.82 | 52.00 | ± | 0.59 |
| 21 | 71.53 | ± | 2.91 | 69.85 | ± | 1.68 | 70.27 | ± | 2.98 | 66.83 | ± | 0.87 |
