# Supplementary material for: Neonatal inflammatory pain and systemic inflammatory responses as possible environmental factors in the development of autism spectrum disorder of juvenile rats
Source: J Neuroinflammation. 2016 May 16;13:109. doi: 10.1186/s12974-016-0575-x (PMC4867541; doi:10.1186/s12974-016-0575-x)
Supplement: Additional file 4: Table S2. — Bouts of home cage behavior. (DOC 69 kb) [file 12974_2016_575_MOESM4_ESM.doc]

**Supplemental Table 2. Bouts of home cage behavior**

| **Group** | **Behavior** | **Control** | | | **Formalin** | | | **Significance** |
| --- | --- | --- | --- | --- | --- | --- | --- | --- |
| **Walk** | Turn | 923.42 | ± | 132.24 | 2572.21 | ± | 412.97 | S |
| **Rest** | Stationary | 222.31 | ± | 49.95 | 309.63 | ± | 93.42 | NS |
|  | Pause | 153.55 | ± | 32.41 | 69.00 | ± | 28.12 | S |
|  | Remain Low | 1023.24 | ± | 201.32 | 3921.14 | ± | 823.11 | S |
|  | Remain Rear Up | 203.34 | ± | 70.12 | 60.89 | ± | 21.29 | NS |
|  | Remain Partially Reared | 1022.24 | ± | 399.14 | 490.42 | ± | 104.23 | NS |
|  | Into Sleep | 24.00 | ± | 10.29 | 79.50 | ± | 15.19 | S |
|  | Into Awaken | 9.42 | ± | 3.23 | 90.14 | ± | 12.00 | S |
| **Repetitive behavior** | Self groom | 162.32 | ± | 27.13 | 151.14 | ± | 38.42 | NS |
|  | Repetitive Jumping | 3.21 | ± | 1.99 | 2.87 | ± | 1.00 | NS |
| **Olfactory activities** | Sniff | 201.42 | ± | 20.14 | 492.10 | ± | 98.33 | S |
|  | Dig | 12.11 | ± | 0.09 | 0.87 | ± | 0.00 | S |
| **Relaxation** | Stretch Body | 327.12 | ± | 128.57 | 480.00 | ± | 172.12 | NS |
| **Uncontrolled muscle contraction** | Twitch | 189.12 | ± | 32.14 | 252.12 | ± | 87.42 | NS |
| **Controlled Movement** | Come Down | 19.25 | ± | 3.99 | 14.21 | ± | 3.23 | NS |
|  | Come Down From Partially Reared | 1098.00 | ± | 298.47 | 1006.88 | ± | 187.83 | NS |
|  | Come Down To Partially Reared | 173.12 | ± | 63.21 | 105.38 | ± | 35.98 | NS |
|  | Rear Up | 17.21 | ± | 3.82 | 9.13 | ± | 2.99 | NS |
|  | Rear up Partially | 1082.34 | ± | 299.14 | 839.75 | ± | 171.43 | NS |

Data were from 6-hr monitoring during night. Age: P21; received saline or formalin during P3 to P5, once a day for three days. N = 16 in control and formalin group, respectively. S: Significant difference at *P*< 0.05; NS: Not significant. Values are shown as the mean±SEM. Unpaired Student’s t-test was used for all comparisons.
