# Supplementary material for: Neonatal inflammatory pain and systemic inflammatory responses as possible environmental factors in the development of autism spectrum disorder of juvenile rats
Source: J Neuroinflammation. 2016 May 16;13:109. doi: 10.1186/s12974-016-0575-x (PMC4867541; doi:10.1186/s12974-016-0575-x)
Supplement: Additional file 5: Table S3. — Time spent in home cage behavior (min). (DOC 49 kb) [file 12974_2016_575_MOESM5_ESM.doc]

**Supplemental Table 3. Time spent in home cage b**ehavior (min)

| **Group** | **Behavior** | **Control** | | | **Formalin** | | | **Significance** |
| --- | --- | --- | --- | --- | --- | --- | --- | --- |
| **Walk** | Turn | 5.05 | ± | 1.26 | 16.67 | ± | 3.40 | S |
| **Rest** | Pause | 169.83 | ± | 47.10 | 19.23 | ± | 4.32 | S |
|  | Remain Low | 116.70 | ± | 16.74 | 141.70 | ± | 33.34 | NS |
|  | Remain Rear Up | 0.87 | ± | 0.29 | 3.53 | ± | 0.90 | S |
|  | Remain Partially Reared | 13.45 | ± | 3.47 | 5.60 | ± | 1.03 | S |
|  | Sleep | 63.69 | ± | 12.02 | 136.89 | ± | 22.00 | S |
| **Repetitive behavior** | Self Groom | 32.37 | ± | 1.83 | 65.09 | ± | 10.36 | S |
|  | Repetitive Jumping | 0.01 | ± | 0.00 | 0.02 | ± | 0.00 | S |
| **Olfactory activity** | Sniff | 17.06 | ± | 2.00 | 20.01 | ± | 6.51 | NS |
| **Relaxation** | Stretch Body | 15.39 | ± | 3.51 | 5.05 | ± | 2.06 | S |
| **Uncontrolled muscle contraction** | Twitch | 3.40 | ± | 0.56 | 13.73 | ± | 2.23 | S |
| **Controlled Movement** | Come Down From Partially Reared | 5.35 | ± | 1.45 | 4.56 | ± | 0.72 | NS |
|  | Rear up Partially | 4.72 | ± | 1.12 | 3.34 | ± | 0.50 | NS |

Data were from 6-hr monitoring during night. Age: P21; received saline or formalin during P3 to P5, once a day for three days. N = 16 in control and formalin group, respectively; S: Significant difference at *P*< 0.05; NS: Not significant. Values are shown as the mean±SEM. Unpaired Student’s t-test was used for all comparisons. Some short and unchanged behavioral activities are not shown in this Table.
